# Antisense oligonucleotide and thyroid hormone conjugates for obesity treatment

**DOI:** 10.1038/s41598-017-09598-z

**Published:** 2017-08-24

**Authors:** Yang Cao, Tomoko Matsubara, Can Zhao, Wei Gao, Linxiu Peng, Jinjun Shan, Zhengxia Liu, Fang Yuan, Lingyi Tang, Peixin Li, Zhibin Guan, Zhuyuan Fang, Xiang Lu, Hu Huang, Qin Yang

**Affiliations:** 1 Department of Medicine, Physiology and Biophysics, UC Irvine Diabetes Center, Center for Epigenetics and Metabolism, University of California Irvine, Irvine, California 92697 USA; 2 Department of Kinesiology & Physiology, East Carolina Diabetes and Obesity Institute, East Carolina University, Greenville, North Carolina 27834 USA; 3 Japan Society for the Promotion of Science, Tokyo, 1020083 Japan; 4 Department of Geriatrics, the Second Affiliated Hospital, Nanjing Medical University, Nanjing, 211166 China; 5 Department of Geriatrics, Sir Run Run Shaw Hospital, Nanjing Medical University, Nanjing, 211166 China; 6 Medical Metabolomics Center, Jiangsu Key Laboratory of Pediatric Respiratory Disease, Nanjing University of Chinese Medicine, Nanjing, 210023 China; 7 Department of Cardiology, the First Affiliated Hospital of Nanjing University of Chinese Medicine, Nanjing, 210029 China; 8 Department of Comprehensive Surgery, Medical and Health Center, Beijing Friendship Hospital, Capital Medical University, Beijing, China; 9 Department of Chemistry, University of California, Irvine, 92697 California USA

## Abstract

Using the principle of antibody-drug conjugates that deliver highly potent cytotoxic agents to cancer cells for cancer therapy, we here report the synthesis of antisense-oligonucleotides (ASO) and thyroid hormone T3 conjugates for obesity treatment. ASOs primarily target fat and liver with poor penetrance to other organs. Pharmacological T3 treatment increases energy expenditure and causes weight loss, but is contraindicated for obesity treatment due to systemic effects on multiple organs. We hypothesize that ASO-T3 conjugates may knock down target genes and enrich T3 action in fat and liver. Two established ASOs are tested. Nicotinamide N-methyltransferase (NNMT)-ASO prevents diet-induced obesity in mice. Apolipoprotein B (ApoB)-ASO is an FDA approved drug for treating familial hypercholesterolemia. NNMT-ASO and ApoB-ASO are chemically conjugated with T3 using a non-cleavable sulfo-SMCC linker. Both NNMT-ASO-T3 (NAT3) and ApoB-ASO-T3 (AAT3) enhance thyroid hormone receptor activity. Treating obese mice with NAT3 or AAT3 decreases adiposity and increases lean mass. ASO-T3 enhances white fat browning, decreases genes for fatty acid synthesis in liver, and shows limited effects on T3 target genes in heart and muscle. Furthermore, AAT3 augments LDL cholesterol-lowering effects of ApoB-ASO. Therefore, ASO and hormone/drug conjugation may provide a novel strategy for obesity and hyperlipidemia treatment.

## Introduction

The antibody-drug conjugates (ADCs) or immunoconjugates have emerged as a novel class of drugs for targeted cancer therapy^1, 2^. ADC is a three-component system in which a therapeutic monoclonal antibody (mAb) and a potent small-molecule cytotoxic drug are conjugated through a stable linker. The mAb specifically recognizes the surface antigen/receptor on cancer cells, which blocks signal transduction. At the meantime, the ADC complex is internalized into cancer cells, where the antibody is degraded and the cytotoxic drug is released. This unique design not only significantly decreases the side effects of cytotoxic drugs, but also allows enrichment of cytotoxic drugs in cancer cells^3^. Two ADC drugs brentuximab vedotin and ado-trastuzumab emtansine have been approved by FDA for the treatment of lymphoma and breast cancer^1^. Many ADC drugs are currently on clinical trials for cancer therapy^4^.

Intrigued by the ADC design, we asked whether a similar strategy could be used for obesity treatment by targeting metabolic organs. Since there are no established antibodies suitable for the ADC design, we turned our focus to antisense oligonucleotides (ASO) as a potential replacement for antibodies. ASOs are modified short single-chain DNA molecules that regulate gene expression primarily in liver and fat^5–7^. The ASOs have very poor bioavailability in skeletal and cardiac muscle, brain, pancreas, following systemic administration^6–8^. In fact, significant efforts have been made towards designing ASO modifications to improve the penetrance to organs other than liver and fat^9^. This limitation of ASOs to treat neurological, muscular and cardiac diseases turns to be advantageous to design ASO-drug conjugates to target fat and liver for obesity treatment. Another important consideration for choosing ASOs is that, similar to cellular uptake of ADC, ASO internalization is shown to be largely through endocytosis^10, 11^.

Increasing energy expenditure is a major strategy for obesity treatment. Thyroid hormone T3 is very powerful in increasing basal metabolic rate and inducing browning of white adipose tissue^12, 13^. Weight loss is a major symptom of hyperthyroidism in humans^14^. However, thyroid hormone is contraindicated for obesity treatment due to its systemic effects on brain, heart and muscle causing anxiety, heart failure, and muscle wasting^14^. Since ASOs penetrate these organs very poorly, we hypothesize that ASO-T3 conjugates would minimize the thyroid hormone effects in these organs.

The next question is what linker to use to conjugate ASOs and T3. We chose sulfosuccinimidyl-4-(N-maleimid-omethyl)cyclohexane-1-carboxylate (Sulfo-SMCC), a non-cleavable and membrane impermeable crosslinker, because of its high selectivity, rapid reaction kinetics, and compatibility with aqueous reaction conditions^15^. Sulfo-SMCC contains an amine-reactive N-hydroxysuccinimide (NHS ester) and a sulfhydryl-reactive maleimide group. The maleimide group of Sulfo-SMCC is unusually stable because of the cyclohexane bridge in the spacer arm. This significantly increases ADC drug stability in circulation. Sulfo-SMCC has been widely used in the synthesis of ADCs including FDA-approved ado-trastuzumab emtansine.

We then tested two established ASOs. Nicotinamide N-methyltransferase (NNMT) is a histone methylation modulator that regulates cellular energy expenditure in adipose tissue^16, 17^. NNMT-ASO treatment prevents diet-induced obesity^16^. The FDA-approved drug Mipomersen is an ASO against Apolipoprotein B (ApoB) for treating familiar hypercholesterolemia^5^. Using the concept of ADC design, we synthesized novel compounds by chemically conjugating NNMT-ASO and ApoB-ASO with T3 hormone using sulfo-SMCC linker. We then investigated the effects of the ASO-T3 bioconjugates on obesity.

## Results

### ASO-T3 synthesis and function

**Figure 1:**
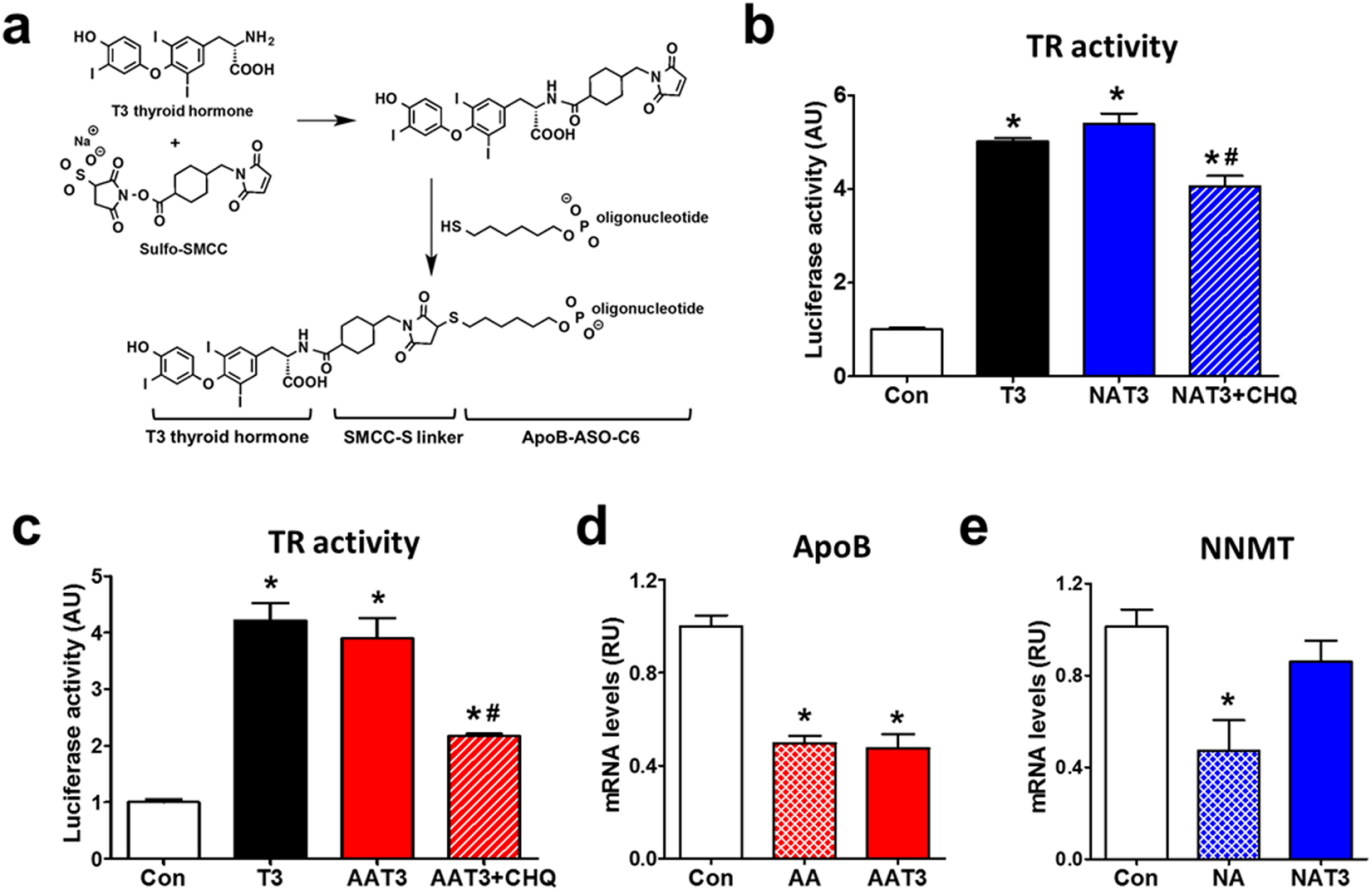
ASO-T3 conjugate structure and function. (**a**) Schematic process of ASO-T3 synthesis. (**b**–**c**) Thyroid hormone receptor reporter activity in HEK293 cells treated with T3, NNMT-ASO-T3 (NAT3) (**b**) or ApoB-ASO-T3 (**c**) with or without the lysosome inhibitor chloroquine (CHQ). (**d**) ApoB mRNA levels in Hepa1-6 hepatoma cells treated with ApoB-ASO (AA) or AAT3. (**e**) NNMT mRNA levels in adipose tissue treated NNMT-ASO (NA) or NAT3. n = 4–6 per group; *p < 0.05 vs controls (Con), ^#^p < 0.05 vs NAT3 or AAT3.

Thyroid hormone T3 was first reacted with sulfo-SMCC to generate maleimide-activated T3. The product was then conjugated to phosphorothioate-modified NNMT-ASO or ApoB-ASO (Fig. 1a). The NNMT-ASO-T3 (NAT3) and ApoB-ASO-T3 (AAT3) products show no detectable free T3 or T3-SMCC (Supplementary Figure 1a–d). The successful conjugation of ASO and T3 was verified by MALDI-TOF MS (Supplementary Figure 2a,b). We then investigated whether ASO-T3 conjugates may maintain the functionality of both ASO and T3. NAT3 and AAT3 activated thyroid hormone receptor reporter (Fig. 1b,c). The effects were attenuated by the treatment of chloroquine, an inhibitor of lysosome acidification, suggesting that lysosome plays an important role in T3 activity of NAT3 and AAT3 (Fig. 1b,c). The ASO activity in ASO-T3 conjugates appears to be target-dependent. AAT3 decreased ApoB expression to the similar degree as ApoB-ASO (AA) in cultured hepatocytes (Fig. 1d), while NAT3 failed to knock down NNMT expression in cultured hepatocytes, adipocytes (not shown) or adipose tissue (Fig. 1e). Since NAT3 has no NNMT-ASO activity and ApoB-ASO is known to have no effects on body weight in animals and humans^18, 19^, we focused on studying the effects of AAT3 and NAT3 on energy expenditure and weight loss.

### Effects of ASO-T3 on obesity

**Figure 2:**
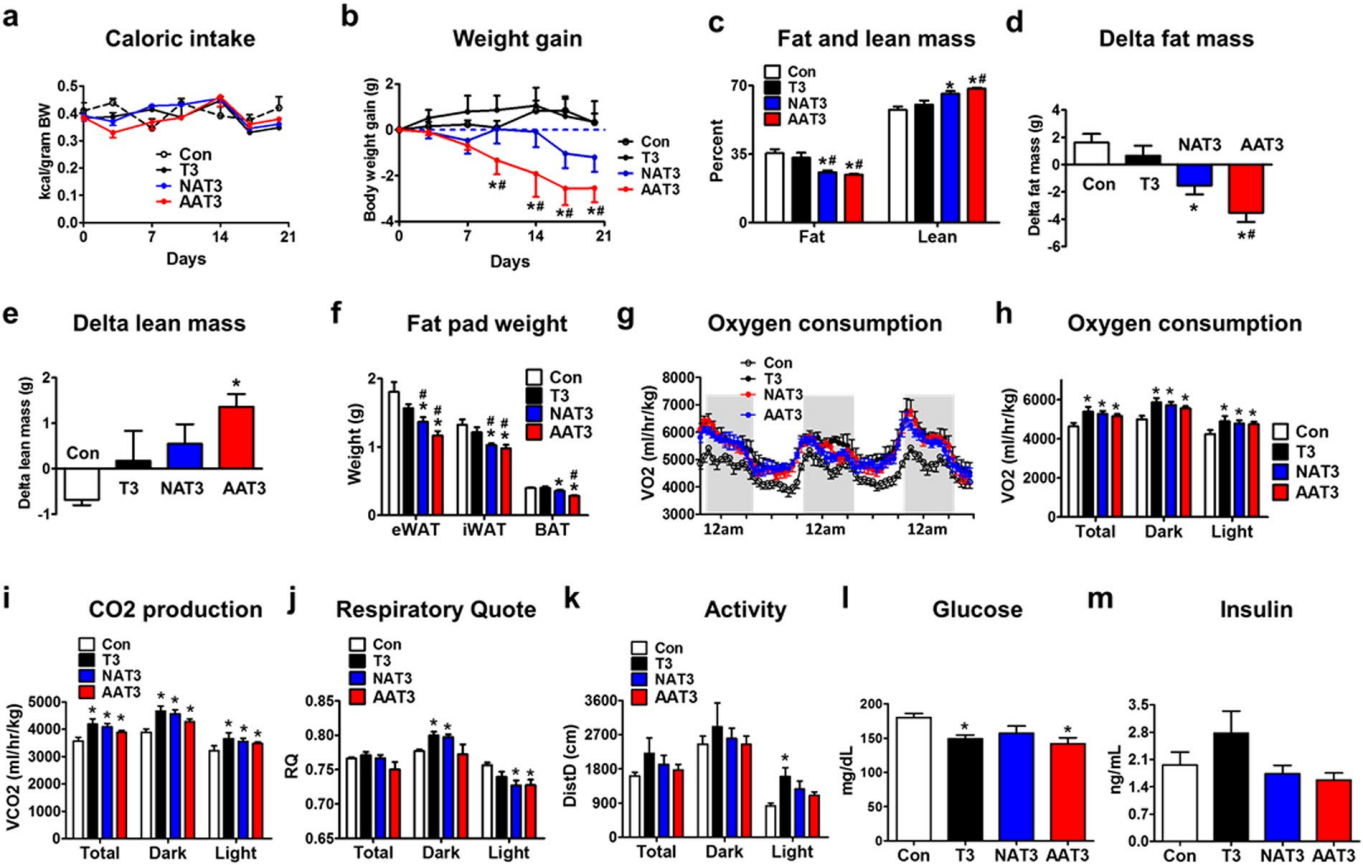
ASO-T3 reduces adiposity in obesity. (**a**–**f**) Caloric intake (**a**), body weight gain (**b**), percent fat and lean mass (**c**), delta fat mass (**d**), delta lean mass (**e**) and weight of epididymal (eWAT), inguinal (iWAT) and brown (BAT) adipose tissue (**f**) in high-fat diet fed mice treated with PBS control, T3, NNMT-ASO-T3 (NAT3) or ApoB-ASO-T3 (AAT3). (**g**–**k**) Metabolic cages studies. Oxygen consumption (**g**,**h**) and CO2 production (**i**) normalized to lean mass, respiratory quote (**j**) and locomotor activity (**k**) in control, T3, NAT3 and AAT3-treated mice. (**l**) Glucose; (**m**) insulin levels in control, T3, NAT3 and AAT3-treated mice. n = 4–6 per group; *p < 0.05 vs controls (Con), ^#^p < 0.05 vs T3.

T3 treatment increased serum T3 by approximately 8 times, while NAT3 and AAT3 did not significantly alter circulating T3 levels (Supplementary Fig. 3a). Interestingly, T3 treatment did not decrease, but increased body weight in mice fed a high fat diet (Supplementary Fig. 3b), despite significantly elevated energy expenditure (Supplementary Fig. 3c). The increased food intake^20, 21^ (Supplementary Fig. 3d) and enhanced nutrient absorption^22^ may contribute to the weight gain in T3-treated mice. We therefore performed pair-feeding experiments. Caloric intake was not different in mice treated with control, T3, NAT3 or AAT3 (Fig. 2a). Despite pair-feeding, T3 treatment did not decrease body weight in three weeks. NAT3 treatment resulted in mild weight loss and AAT3 was a more potent ASO-T3 bioconjugate to induce weight loss (Fig. 2b). NAT3 and AAT3, but not T3 decreased fat mass and increased lean mass (Fig. 2c–e). Consistently, individual depot mass including epididymal fat, inguinal fat and brown fat were all decreased in NAT3 and AAT3-treated mice compared to control and T3-treated mice (Fig. 2f). In the metabolic cage studies, T3, NAT3 and AAT3 all elevated oxygen consumption and CO2 production (Fig. 2g–i). NAT3 and AAT3 decreased respiratory ratio compared to control and T3 treatment during light cycles, indicating increased fat metabolism (Fig. 2j). T3 also significantly increased locomotor activity during the light cycles compared with controls (Fig. 2k), which might be due to anxiety and/or food-seeking behavior. Glucose levels were lower in T3, NAT3 and AAT3-treated mice (Fig. 2l). Insulin levels tended to be higher with T3 treatment, but lower in NAT3 and AAT3 treated mice (Fig. 2m). These results indicate that ASO-T3 conjugates are effective in increasing energy expenditure and treating obesity in mice.

### Effects of ASO-T3 treatment on T3 target gene expression in metabolic organs

**Figure 3:**
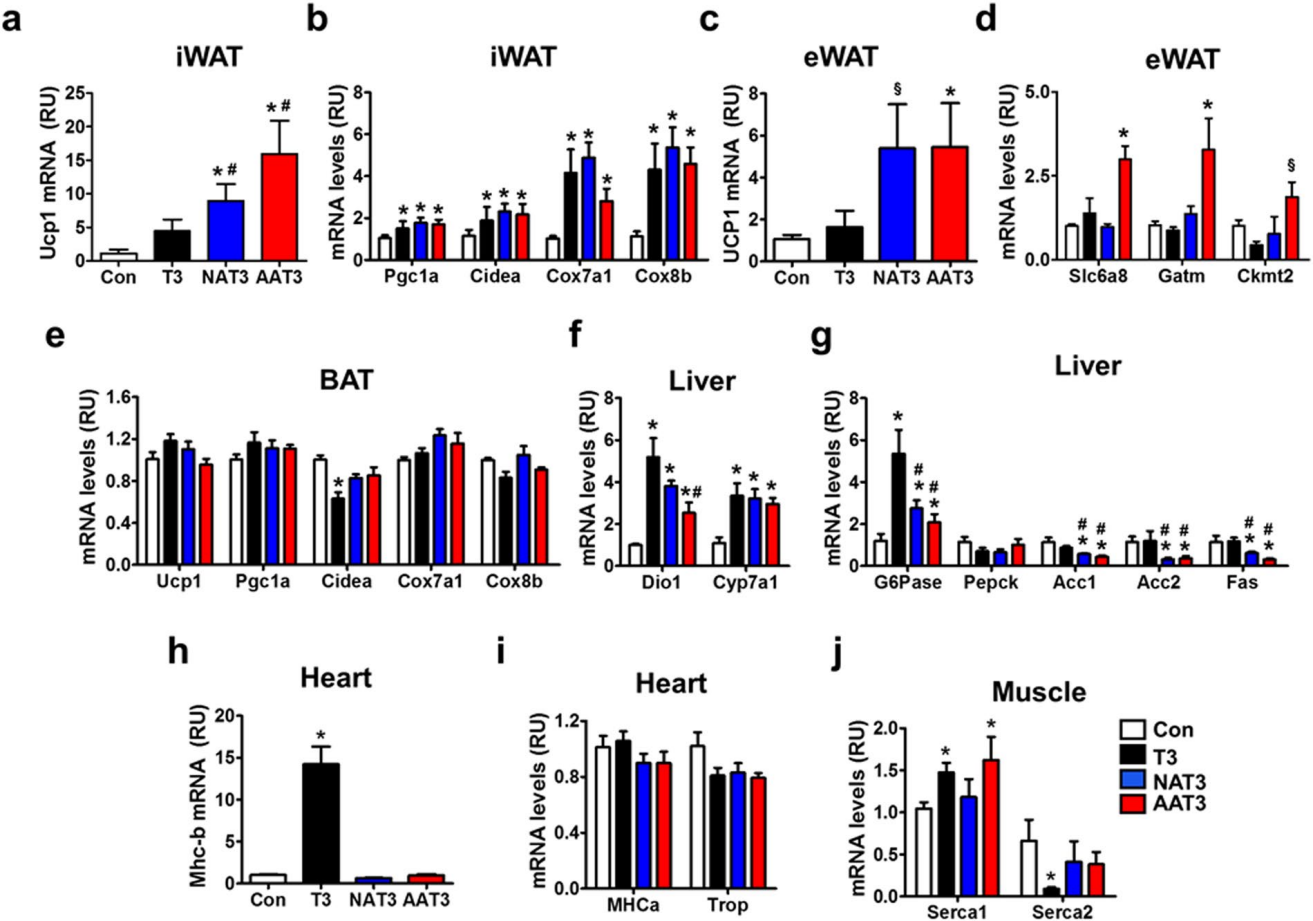
Effects of ASO-T3 on gene expression in metabolic organs. (**a**,**b**) Browning markers (Ucp1, Pgc1a, Cidea, Cox7a1 and Cox8b) in inguinal white adipose tissue (iWAT). (**c**) Ucp1 expression in epididymal white adipose tissue (eWAT). (**d**) Expression of solute carrier family 6 member 8 (Scl6a8), glycine amidinotransferase (Gamt) and creatine kinase mitochondrial 2 (Ckmt2) in eWAT. (**e**) Expression of brown adipose tissue (BAT) gene markers. (**f**) Hepatic expression of thyroid hormone responsive genes deiodinase-1 (Dio1) and cholesterol 7alpha-hydroxylase (Cyp7a1). (**g**) Gluconeogenetic genes (G6pase, Pepck) and fatty acid synthesis genes (Acc1, Acc2 and Fas) in liver. (**h**,**i**) Expression of Mhc-b (**h**) and Mhc-a and Troponin (Trop) (**i**) in heart. (**j**) Expression of Serca1and Searca2a in muscle. NAT3: NNMT-ASO-T3; AAT3: ApoB-ASO-T3. n = 4–6 per group; *p < 0.05 vs control, ^§^p = 0.07 vs control, ^#^p < 0.05 vs T3.

Treatment of T3, NAT3 and AAT3 all induced the expression of browning markers in inguinal white adipose tissue (iWAT) (Fig. 3a,b). NAT3 and AAT3 were stronger in inducing Ucp1 expression comparing with T3 (Fig. 3a). It is recently reported that epididymal white adipose tissue (eWAT) may also participate in thermogenic response via Ucp1-dependent and creatine dependent pathways^23^. Interestingly, NAT3 and AAT3 both elevated Ucp1 expression in eWAT (Fig. 3c). In addition, AAT3 increased expression of Slc6a8, Gatm and Ckmt2, the genes involved in creatine metabolism (Fig. 3d). These results suggest that thermogenic activation in eWAT may also contribute to the increased energy expenditure in ASO-T3-treated mice. In brown adipose tissue (BAT), neither T3, NAT3 nor AAT3 had significant effects on BAT activity (Fig. 3e). In liver, NAT3 and AAT3 appeared to have comparable or lower activity on the thyroid hormone target genes such as Dio1, Cyp7a1 and G6pase, compared with T3 alone (Fig. 3f,g). However, NAT3 and AAT3 had stronger effects on suppressing fatty acid synthesis as evidenced by decreased expression of Acc1, Acc2 and Fas compared with control and T3 treatment (Fig. 3g). In heart, T3 treatment drastically increased the expression of Mhc-b, a marker for cardiac failure, by approximately 13 times compared with controls. NAT3 and AAT3 did not affect Mhc-b expression (Fig. 3h). Neither T3, NAT3 nor AAT3 affected the expression of Mhc-a or troponin (Fig. 3i). In skeleton muscle, thyroid hormone differentially regulates the isoforms of the Ca^2+^-ATPase (Serca) by increasing the fast-muscle isoform (Serca1) and suppressing the slow-muscle isoform (Serca2a)^24^. Consistently, we found that T3, but not NAT3 increased Serca1. For unknown reasons, AAT3 also increased Serca1 expression. T3 strongly suppressed Serca2a expression while NAT3 and AAT3 had no effects (Fig. 3j). These results indicate that ASO-T3 selectively regulates thyroid hormone target genes in adipose tissue and liver.

### ApoB-ASO and T3 conjugates improve hypercholesterolemia

**Figure 4:**
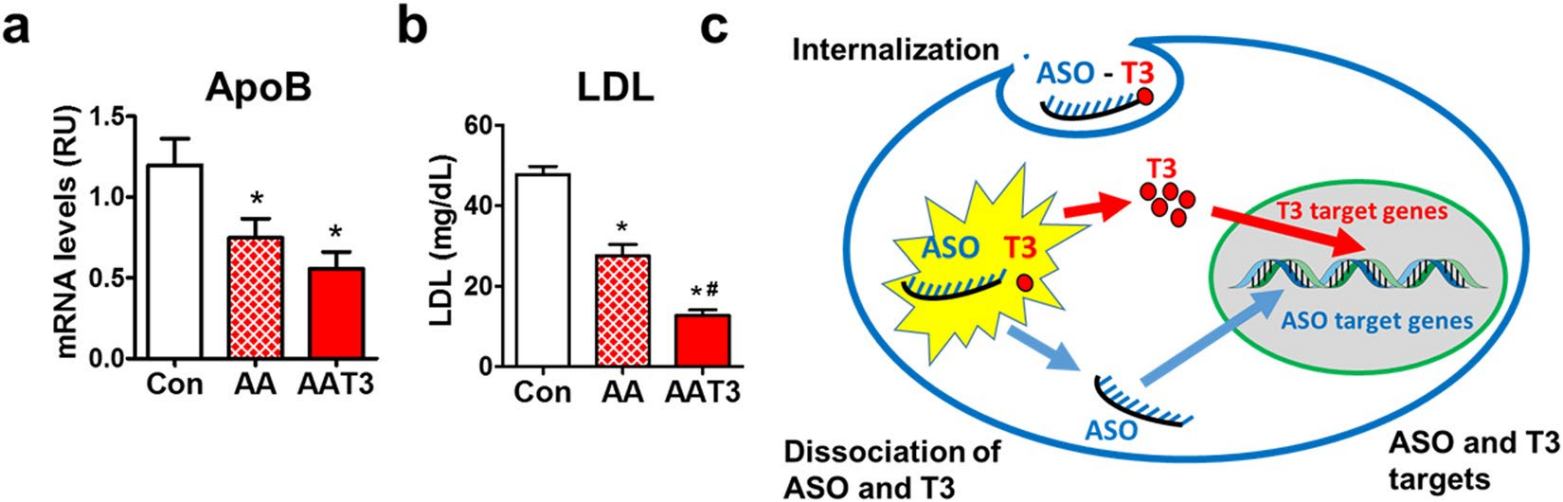
Effects of ApoB-ASO-T3 (AAT3) on LDL levels. (**a**) Apolipoprotein B (ApoB) mRNA levels in liver. (**b**) Serum LDL levels. AA: ApoB-ASO. n = 5–6 per group; *p < 0.05 vs controls, ^#^p < 0.05 vs AA. (**c**) Schematic working model for ASO-T3 action. ASO-T3 is internalized and processed in cells. ASO knocks down the target gene and T3 exerts its biological functions by activating nuclear thyroid hormone receptor.

AAT3 maintained the ASO activity in cultured hepatocytes (Fig. 1d). In mice treated with AAT3, the ApoB expression was slightly lower than that in AA-treated mice (Fig. 4a). The serum LDL levels was significantly decreased in AAT3-treated mice compared that in AA-treated mice (Fig. 4b). The results suggest potential synergistic effects of AA and T3 on lowing LDL levels in obesity.

## Discussion

Despite our better understanding of the pathophysiology of obesity, the drug development for obesity treatment is lagging behind. Increasing energy expenditure to overcome energy intake is a fundamental strategy for obesity drug development^25^. Many drugs such as thyroid hormone and adrenergic receptor agonists are quite potent in increasing energy expenditure^26–28^. However, the systemic effects of these drugs prohibit the use for obesity treatment. The situation is similar to the cytotoxic drugs for cancer therapy. Although effective in killing cancer cells, cytotoxic drugs also significantly damage normal tissues. The art of ADC drug design brings a therapeutic revolution in cancer treatment, allowing targeted delivery of cytotoxic drugs to cancer cells. Using the concept of ADC design and taking advantages of adipose and hepatic targeting effects of ASOs, we successfully developed ASO-T3 conjugates. Recently, a hybrid hormone of glucagon-T3 conjugation has been reported to selectively target adipose tissue and liver leading to remarkable weight loss and improvement of metabolic syndrome in obese mice^29^. Therefore, the novel drug design to induce adipose and hepatic hyperthyroidism may lead to a new direction for drug development for the treatment of obesity and metabolic syndrome.

The ideal outcome of ASO-T3 design is to retain both ASO and T3 activity so that ASO can knock down the target gene and T3 can activate thyroid hormone receptors in fat and liver. The goal is to achieve synergistic effects of ASO and T3 on improving glucose, lipid and energy metabolism. However, conjugation of small molecules to ASOs may affect ASO stability and activity^29, 30^. For the two ASO-T3 conjugates we tested, only ApoB-ASO-T3 maintains the ASO activity, while NNMT-ASO-T3 fails to knock down NNMT. Therefore, careful ASO selection and testing are critical to successful ASO-T3 conjugation.

Although both T3 and ASO-T3 increase energy expenditure, only ASO-T3 treated mice show reduced body weight and adiposity. Different from weight loss in hyperthyroid humans, euthyroid mice treated with T3 systemically do not lose, but gain weight. This is largely due to the direct effects of T3 on hypothalamus to stimulate food intake and effects on intestine to increase nutrient absorption in mice^20–22^. Despite pair feeding, energy expenditure and absorption appear to be balanced in T3-treated mice. In ASO-T3-treated mice, however, circulating T3 is not elevated. Thus, there is no systemic T3 action to compensate the increased energy expenditure, leading to weight loss and reduced adiposity.

One important consideration to use ASOs as the tissue-homing vector is that ASOs, similar to the antibodies in ADC, are internalized into tissue cells by endocytosis *in vivo* ^10, 11^ (Fig. 4c). The physiological endocytosis process does not damage the integrity of cellular plasma membrane and therefore less toxic. Intracellularly, ASOs are either degraded in lysosomes directly or hybridized with RNAs and degraded by nucleases^10, 31, 32^. Inhibition of lysosome acidification with chloroquine decreased, but did not completely abolish T3 activity (Fig. 1b,c), suggesting that both pathways might be involved in the ASO and T3 dissociation in cultured cells.

Although effective in treating obesity, the current ASO-T3 drug design could be further improved to increase the T3 payload and delivery efficiency. In the ADC design, many chemotherapy drug molecules are conjugated to the cysteine or lysine residues at different positions of one antibody molecule, which maximizes cytotoxic effects^3^. Since ASOs are much smaller molecules compared with antibodies, one ASO is conjugated to one T3 molecule. Whether adding multiple T3 to one ASO molecule is feasible remains to be tested. In addition, the activity of ASO-T3 on thyroid hormone receptor reporter is about 1/1000 compared with that of T3 in our *in vitro* assays (Fig. 1b,c). Approaches such as testing different conjugation linkers^33^ may increase the dissociation between ASO and T3 to enhance the biological activity of both ASO and T3. Finally, although liver is the primary target organ for ASOs, the preference of ASO accumulation in liver is the non-parenchymal cells rather than hepatocytes^34–36^. This may at least partially explain the relatively lower expression of Dio1, a very sensitive marker for hepatic thyroid hormone activity^37^, in ASO-T3 treated mice compared with that in T3-treated mice. Future studies to add modifications such as triantennary N-acetyl galactosamine^34–36^ or adipose-targeting peptide^38, 39^ to ASOs may increase the bioavailability of ASO-T3 in liver and adipose tissue. Despite these limitations, the current drug design provides a prototype model for a new class of drugs for treating obesity.

In summary, using the principle of ADC, we have successfully designed ASO-T3 conjugates and proved the concept that ASO-T3 may represent a novel strategy for drug development for obesity treatment. Importantly, ApoB-ASO (Mipomersen) is an FDA-approved drug with established safety profiles for treating familiar hypercholesterolemia. ApoB-ASO-T3 conjugate may potentially be used for general obese patients with hypercholesterolemia.

## Materials and Methods

### ASO-T3 synthesis

Phosphorothioate-modified ASOs^16, 19^ (mouse NNMT-ASO: 5-GAAATGAACCAGCAGGCCTT-3 and mouse ApoB-ASO: 5-GTCCCTGAAGATGTCAATGC-3) with a 5′- thiol and C6 amino linker were synthesized by Bio-Synthesis (Lewisville, Texas). Thyroid hormone T3 was first reacted with sulfo-SMCC to generate maleimide-activated T3. After reversed-phase high-performance liquid chromatography (RP-HPLC) purification, excessive T3-SMCC is reacted with the thiol-functionalized oligonucleotides to generate ASO-T3 conjugates. The conjugates were purified by RP-HPLC and characterized by MALDI-TOF MS.

### Thyroid hormone receptor activity

HEK293T cells were maintained in Dulbecco’s modified Eagle’s medium supplemented with L-glutamine, 10% steroid hormone-depleted fetal calf serum, penicillin and streptomycin. Transient transfections were performed in 6-well plates of subconfluent cells. Two-step transfections were performed because no luciferase activity could be measured when the plasmids were co-transfected with ASOs (not shown). Plasmids including thyroid hormone receptor alpha (100 ng), thyroid hormone receptor beta (100 ng), DR4-luciferase thyroid hormone receptor (100 ng) and PRL-TK (10 ng) were first transfected to HEK293T cells using FuGENE HD transfection reagent (Roche)^40^. Twelve hours later, ASO-T3 (1 μM) was transfected to the cells. Luciferase activity was measured 36 hours after the ASO-T3 transfection. The DR4-driven Firefly luciferase activity was normalized to PRL-TK-driven Renilla luciferase activity. Chloroquine (100 μM) was added to inhibit lysosome acidification 6 hours prior to luciferase activity measurements. T3 (1 nM) was added 24 hours prior to cell harvesting for positive controls of thyroid hormone reporter activity.

### ASO-T3 treatment in mice

Wild type C57BL/6 mice were purchased from the Jackson Laboratory and housed in a 12-hour light/dark cycle (lights on 7 am-7 pm). The mice were first treated with a high fat diet (Research Diets D12331, 5.56 kcal/gm) for 12 weeks. An MRI was performed for the baseline body composition. The mice were then treated with PBS control, T3 (200 μg/kg) or ASO-T3 (25 mg/kg) twice a week. The metabolic cage studies were started from day 7 when there was no difference in body weight. A repeat MRI was performed three weeks after the treatment. Mouse studies were conducted in accordance with federal guidelines and were approved by the Institutional Animal Care and Use Committee of University of California Irvine and East Carolina University.

### Serum T3 measurements

Serum T3 was measured using liquid chromatography-tandem mass spectrometry (LC-MS/MS)^41, 42^. Eighty-microliter serum from mice treated with T3 or ASO-T3 for two hours was mixed with ascorbic acid, citric acid and dithiothreitol (25 μg/μl) to prevent the potential conversions of thyroid hormones. After one-hour incubation on ice, 640 μl urea was added and the solution was incubated at 50 °C for one hour. The samples were then subjected to solid phase extraction before the injection to the LC-MS/MS. Separation was achieved using GP-Phenyl (3 μm, 2.1 × 100 mm). The gradient mobile phase consisted of 0.1% formic acid in water (A) and acetonitrile (B) and the flow rate was set at 0.2 mL/min. Mass spectrometry detection was operated on TSQ Vantage Triple-Stage Quadrupole Mass Spectrometer, Spray voltage:3.2 KV, Vaporizer temperature:200 °C, Sheath gas pressure:45 arb, Aux gas pressure:15 arb, Capillary temperature:270 °C. The quantification was performed by the selected reaction monitoring (SRM) in the positive ionization mode and the parameters is m/z 651.517 → 508.000 for T3 (CE of 22 eV; S-lens at 161 V), 461.095 → 285.056 for IS (CE of 19 eV; S-lens at 88 V).

### Body composition

Body composition was analyzed in mice treated with control PBS, T3, NNMT-ASO-T3 or ApoB-ASO-T3 for 3 weeks using an EchoMRI 3-in-1 instrument (Echo Medical Systems, Houston, TX).

### Metabolic cages

Energy expenditure was evaluated using TSE PhenoMaster System. HFD-fed mice were treated with Control PBS, T3, NNMT-ASO-T3 or ApoB-ASO-T3 for 7 days. The mice were acclimated in the metabolic cages for 2 days before the experiments. CO2 production and O2 consumption were collected every 60 min for each mouse during a period of 3–4 days. The levels were normalized to the lean mass. Food intake and activity were measured at regular intervals.

### Serum glucose, insulin and LDL measurements

Glucose levels were measured using the glucose colorimetry assay (Cayman Chemicals). Insulin was measured with Ultrasensitive Insulin ELISA kit (Crystal Chem). LDL was measured using LDL Assay kit from BioAssay Systems.

### RNA extraction and quantitative PCR

Adipose tissue and liver RNA was extracted using RNeasy Mini Kit (Qiagen). RNA from heart and muscle was extracted using RNeasy Fibrous Tissue Mini kit (Qiagen). Hepa1-6 mouse hepatoma cells were transfected with ASO and ASO-T3. RNA was extracted 48 hours after transfection. SYBR Realtime PCR were performed as described previously^43^. The primers are listed in Supplementary Table-1.

### Statistical test

All data are expressed as mean ± s.e.m. Analyses of variance were performed followed by Bonferroni-Holm posthoc tests for multiple comparisons. Statistical significance is assumed for p < 0.05.

## Electronic supplementary material

Supplementary info
