# Supplementary material for: Antisense oligonucleotide and thyroid hormone conjugates for obesity treatment
Source: Sci Rep. 2017 Aug 24;7:9307. doi: 10.1038/s41598-017-09598-z (PMC5571112; doi:10.1038/s41598-017-09598-z)
Supplement: Supplementary file 1 — Supplementary info [file 41598_2017_9598_MOESM1_ESM.pdf]

Antisense oligonucleotide and thyroid hormone conjugates for obesity treatment

Yang Cao[1†], Tomoko Matsubara[2,3†], Can Zhao[1,4], Wei Gao[1,5], Linxiu Peng[6], Jinjun Shan[6], Zhengxia Liu[1,4], Fang Yuan[1,7], Lingyi Tang[1,7], Peixin Li[2,8], Zhibin Guan[9], Zhuyuan Fang[7], Xiang Lu[5], Hu Huang[2*], Qin Yang[1*]

[1] Department of Medicine, Physiology and Biophysics, UC Irvine Diabetes Center, Center for Epigenetics and Metabolism, University of California Irvine, Irvine, California 92697, USA.

[2] Department of Kinesiology & Physiology, East Carolina Diabetes and Obesity Institute, East Carolina University.

[3] Japan Society for the Promotion of Science, Tokyo 1020083, Japan.

[4] Department of Geriatrics, the Second Affiliated Hospital, Nanjing Medical University, Nanjing 211166, China.

[5] Department of Geriatrics, Sir Run Run Shaw Hospital, Nanjing Medical University, Nanjing 211166, China.

[6] Medical Metabolomics Center, Jiangsu Key Laboratory of Pediatric Respiratory Disease, Nanjing University of Chinese Medicine, Nanjing 210023, China.

[7] Department of Cardiology, the First Affiliated Hospital of Nanjing University of Chinese Medicine, Nanjing 210029, China.

[8] Department of Comprehensive Surgery， Medical and Health Center, Beijing Friendship Hospital, Capital Medical University, Beijing, China.

[9] Department of Chemistry, University of California, Irvine.

[†]Y.C. and T.M. contributed equally to this work.

***Address Correspondence to**: Q.Y. (qin.yang@uci.edu) or H.H. (huangh@ecu.edu).

Supplementary Information

Supplementary Figures and Legends

Supplementary Table

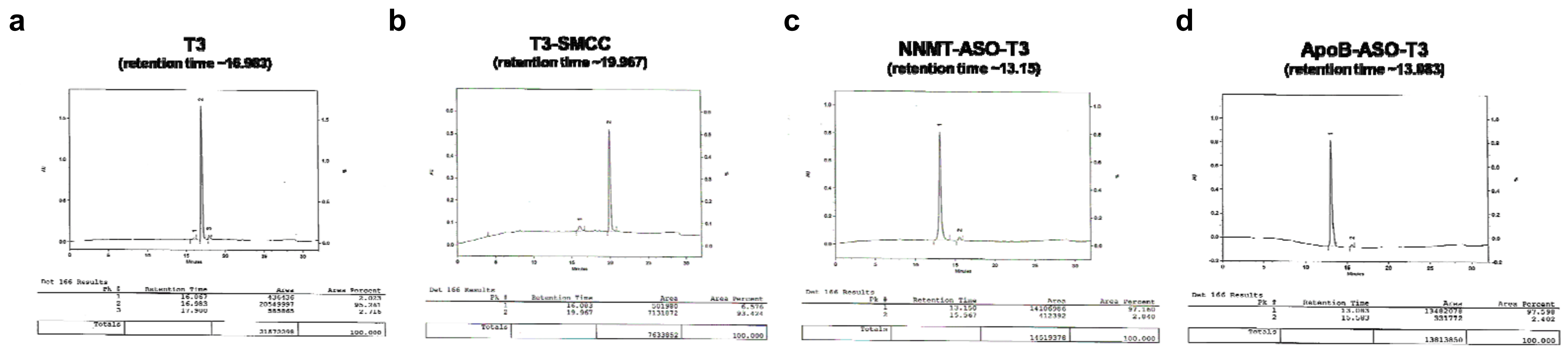

Supplementary Figure 1. HPLC chromatograph for ASO-T3 synthesis. HPLC chromatograph was recorded with the same condition for T3 with retention time 16.983 min (A), T3-SMCC with retention time 19.967 min (B), NNMT-ASO-T3 with retention time 13.15 min (C) and ApoB-ASO-T3 with retention time 13.083 (D). T3 thyroid and T3-SMCC were not observed at ~19.983 and ~19.967 in chromatographs of NNMT-ASO-T3 and ApoB-ASO-T3.

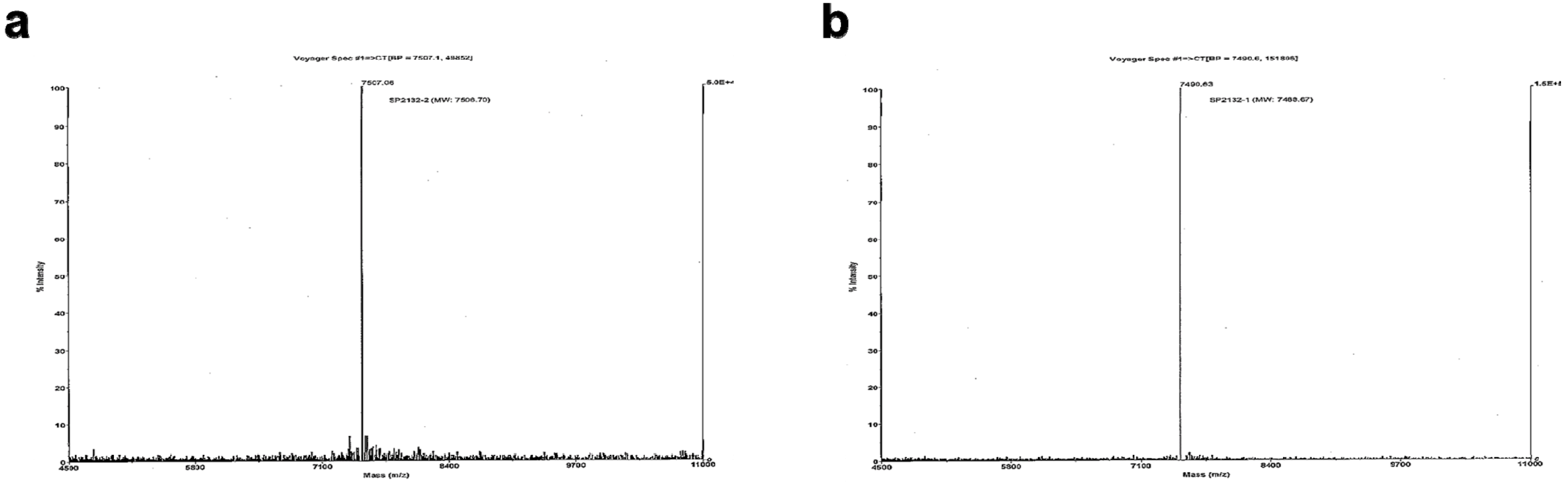

Supplementary Figure 2. MALDI-TOF MS spectra for ASO-T3. (A) NNMT-ASO-T3, molecular weight 7506.70, MALDI-TOF MS 7507.06. (B) ApoB-ASO-T3, molecular weight 7488.67, MALDI-TOF MS 7490.63.

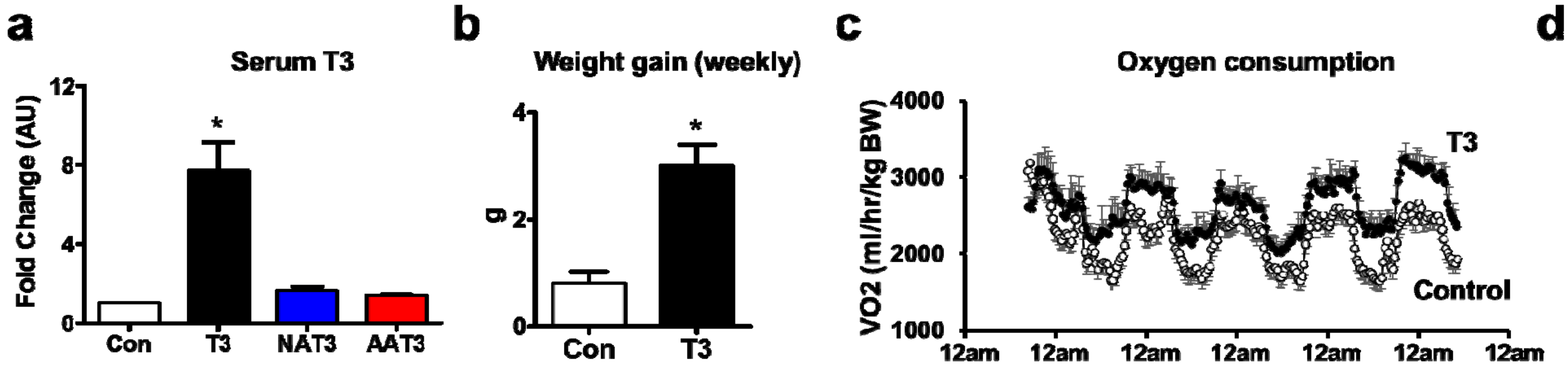

Supplementary Figure 3. Metabolic effects of thyroid hormone T3 in mice. (A) Serum T3 levels in mice treated with control PBS, T3, NNMT-ASO-T3 (NAT3) or ApoB-ASO-T3 (AAT3). (B) Weekly body weight gain with T3 treatment. (C) Oxygen consumption in mice treated with PBS controls or T3 for two weeks. (D) Daily food intake in mice treated with PBS or T3. n=4-6 per group, *p<0.05.

Table 1: Real time PCR primers

Gene	Forward Primer	Reverse Primer
Nnmt	5'-TGTGCAGAAAACGAGATCCTC-3'	5'-AGTTCTCCTTTTACAGCACCCA-3'
ApoB	5'-AAGCACCTCCGAAATGACTGT-3'	5'-CTCCAGCTCTACCTTACAGTTGA-3'
Ucp1	5'-AGGCTTCCAGTACCATTAGGT-3'	5'-CTGAGTGAGGCAAAGCTGATTT-3'
Pgc1a	5'-TATGGAGTGACATAGAGTGTGCT-3'	5'-CCACTTCAATCCACCCAGAAAG-3'
Cidea	5'-TGACATTCATGGGATTGCAGAC-3'	5'-GGCCAGTTGTGATGACTAAGAC-3'
Cox7a1	5'-GCTCTGGTCCGGTCTTTTAGC-3'	5'-GTACTGGGAGGTCATTGTCGG-3'
Cox8b	5'-TGTGGGGATCTCAGCCATAGT-3'	5'-AGTGGGCTAAGACCCATCCTG-3'
Dio1	5'-GCTGAAGCGGCTTGTGATATT-3'	5'-GTTGTCAGGGGCGAATCGG-3'
Cyp7a1	5'-GGGATTGCTGTGGTAGTGAGC-3'	5'-GGTATGGAATCAACCCGTTGTC-3'
G6Pase	5'-CGACTCGCTATCTCCAAGTGA-3'	5'-GTTGAACCAGTCTCCGACCA-3'
Pepck	5'-CTGCATAACGGTCTGGACTTC -3'	5'- CAGCAACTGCCCGTACTCC-3'
Acc1	5'-TGTACAAGCAGTGTGGGCTGGCT-3'	5'-CCACATGGCCTGGCTTGGAGGG-3'
Acc2	5'- CGCTCACCAACAGTAAGGTGG-3'	5'- GCTTGGCAGGGAGTTCCTC-3'
Fas	5'-GCTGCGGAAACTTCAGGAAAT-3'	5'-AGAGACGTGTCACTCCTGGACTT-3'
Mhc-b	5'-GCCCAGTACCTCCGAAAGTC-3'	5'-GCCTTAACATACTCCTCCTTGTC-3'
Mhc-a	5'-GAGCAAGGCCGAGGAGACGCAGCGT-3'	5'-GAGCCTTCTCGTCCAGCTGCCGG-3'
Troponin-I	5'-TCTGCCAACTACCGAGCCTAT-3'	5'-CTCTTCTGCCTCTCGTTCCAT-3'
Serca1	5'-TGTTTGTCCTATTTCGGGGTG-3'	5'-AATCCGCACAAGCAGGTCTTC-3'
Serca2a	5'-GAGAACGCTCACACAAAGACC-3'	5'-CAATTCGTTGGAGCCCCAT-3'
Slc6a8	5'-TGCATATCTCCAAGGTGGCAG-3'	5'-CTACAAACTGGCTGTCCAGA -3'
Gatm	5'-GACCTGGTCTTGTGCTCTCC -3'	5'-GGGATGACTGGTGTTGGAGG -3'
Ckmt2	5'-GCATGGTGGCTGGTGATGAG-3'	5'-AAACTGCCCGTGAGTAATCTTG -3'
Tbp	5'-CCCTATCACTCCTGCCACAC-3'	5'-ACGAAGTGCAATGGTCTTTAGG-3'
